# The order of expression is a key factor in the production of active transglutaminase in *Escherichia coli *by co-expression with its pro-peptide

**DOI:** 10.1186/1475-2859-10-112

**Published:** 2011-12-23

**Authors:** Song Liu, Dongxu Zhang, Miao Wang, Wenjing Cui, Kangkang Chen, Guocheng Du, Jian Chen, Zhemin Zhou

**Affiliations:** 1 Key Laboratory of Industrial Biotechnology, Ministry of Education, School of Biotechnology, Jiangnan University, Lihu Avenue, Wuxi, China; 2 State Key Laboratory of Food Science and Technology, Jiangnan University, Lihu Avenue, Wuxi, China; 3 School of Food Science and Technology, Jiangnan University, Lihu Avenue, Wuxi, China

**Keywords:** *Streptomyces hygroscopicus*, transglutaminase, pro-peptide, co-expression, *Escherichia coli*

## Abstract

**Background:**

*Streptomyces *transglutaminase (TGase) is naturally synthesized as zymogen (pro-TGase), which is then processed to produce active enzyme by the removal of its N-terminal pro-peptide. This pro-peptide is found to be essential for overexpression of soluble TGase in *E. coli*. However, expression of pro-TGase by *E. coli *requires protease-mediated activation *in vitro*. In this study, we developed a novel co- expression method for the direct production of active TGase in *E. coli*.

**Results:**

A TGase from *S. hygroscopicus *was expressed in *E. coli *only after fusing with the pelB signal peptide, but fusion with the signal peptide induced insoluble enzyme. Therefore, alternative protocol was designed by co-expressing the TGase and its pro-peptide as independent polypeptides under a single T7 promoter using vector pET-22b(+). Although the pro-peptide was co-expressed, the TGase fused without the signal peptide was undetectable in both soluble and insoluble fractions of the recombinant cells. Similarly, when both genes were expressed in the order of the TGase and the pro-peptide, the solubility of TGase fused with the signal peptide was not improved by the co-expression with its pro-peptide. Interestingly, active TGase was only produced by the cells in which the pro-peptide and the TGase were fused with the signal peptide and sequentially expressed. The purified recombinant and native TGase shared the similar catalytic properties.

**Conclusions:**

Our results indicated that the pro-peptide can assist correct folding of the TGase inter-molecularly in *E. coli*, and expression of pro-peptide prior to that of TGase was essential for the production of active TGase. The co-expression strategy based on optimizing the order of gene expression could be useful for the expression of other functional proteins that are synthesized as a precursor.

## Background

Transglutaminase (EC 2.3.2.13, TGase) catalyzes crosslinking between the γ-carboxyamide groups of glutamine residues (acyl donors) and a variety of primary amines (acyl acceptors) in many proteins [1]. In the absence of primary amines, H_2_O can act as an acyl acceptor, resulting in the deamidation of glutamine residues [1]. Multifunctional TGases are widely found in mammals [2], plants [3], and microorganisms [1]. Since the first microbial TGase was discovered in *Streptomyces mobaraensis *[4], and many other TGase-producing microbial strains have been identified [5]. The *Streptomyces *TGase has been widely applied in the food industry to improve the functional properties of food products [1]. Recent research suggests that TGase-mediated crosslinking also has great potential for applications in tissue engineering, textiles and leather processing, biotechnological research, and other non-food uses [6]. The development of an efficient and easy-to-use expression system for the production of TGase is therefore highly desirable.

Previous attempts have been made to express *Streptomyces *TGase in *S. lividans*, *Escherichia coli*, *Corynebactetium glutamicum*, and methylotropic yeasts [5]. Among these studies, expression of TGase in *E. coli *has received extensive attention due to its ease of culture and genetic manipulation. Furthermore, *E. coli *expression system is the most suitable screening platform for directed evolution. *Streptomyces *TGase is naturally synthesized as pro-TGase, which is then processed by the removal of its N-terminal pro-peptide to produce active TGase [7,8]. Therefore, three strategies have been used for the expression of the microbial TGase in *E. coli*: (i) the direct expression of TGase fused or not fused to an additional peptide; (ii) the expression of pro-TGase followed by processing to TGase *in vitro*; and (iii) the co-expression of pro-TGase with the activating protease. The first strategy often leads to a low-level of the protein expression or the inclusion body formation [9,10]. The second strategy produces a large amount of soluble pro-TGase [11,12] that can be converted into an active TGase *in vitro *by adding exogenous proteases [13]. These results suggest that the covalently linked pro-peptide could facilitate TGase solubility in *E. coli*. In the third strategy, the active TGase is produced directly by *E. coli*, without activation of pro-TGase *in vitro *[14]. However, the protein degradation mediated by co-expressed protease is obviously unfavorable for the application of TGase that catalyze the crosslinking of proteins [1] and also a negative impact on TGase production [15].

To avoid the side effects of the protease and to produce active TGase, Yurimoto et al. [16] established a new co-expression system in which the gene of *Streptomyces *TGase and its pro-peptide were integrated into the genome of a methylotrophic yeast at two different loci. Although the glycosylation of the yeast diminishes the activity of the enzyme, the successful expression of active TGase using this system suggests that the pro-peptide assists TGase folding in an inter-molecular manner. However, this work pointed out that similar strategies pursued in bacterial expression system could not produce active TGase [16]. In this study, we developed a novel expression method for the direct production of active TGase in *E. coli*, based on the co-expression of TGase and its pro-peptide as independent polypeptides under a single T7 promoter. Our results indicated that the expression of pro-peptide prior to that of TGase was essential for the active TGase production in *E. coli*.

## Results and Discussion

### Expression of TGase and pro-TGase

**Figure 1:**
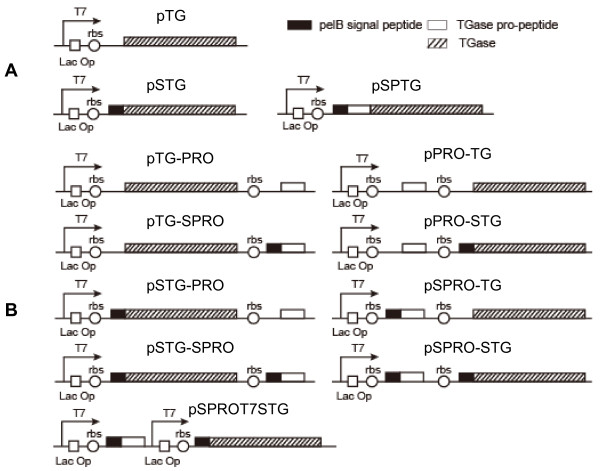
**Genetic organization for the construction of a set of vectors**. (A) Single expression vectors. (B) Co-expression vectors.

**Figure 2:**
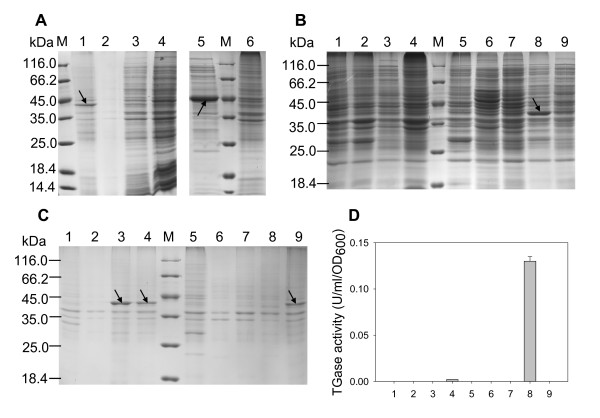
**Expression of pro-TGase and TGase in *E. coli***. (A) SDS-PAGE analysis of TGase expression using single expression vectors. 1, 2: insoluble fractions of *E. coli *cells harboring pSTG and pTG, respectively; 3, 4: intracellular soluble fractions of cells harboring pSTG and pTG, respectively. 5, 6: supernatant and whole cell extracts of *E. coli *harboring pSPTG, respectively. (B) SDS-PAGE analysis of TGase expression using co-expression constructs (intracellular soluble fractions). (C) SDS-PAGE analysis of TGase expression using co-expression vectors (insoluble fractions). (D) Intracellular TGase activity of *E. coli *cells harboring the co-expression constructs. 1-9: *E. coli *cells harboring pTG-PRO, pTG-SPRO, pSTG-PRO, pSTG-SPRO, pPRO-TG, pPRO-STG, pSPRO-TG, pSPRO-STG, and pSPROT7STG, respectively. The bands of TGase and pro-TGase are indicated by downward and upward arrows, respectively.

*Streptomyces *TGase is synthesized as inactive pro-TGase, which is then processed by the removal of its N-terminal pro-peptide to produce active TGase [7,8]. Many studies have shown that the pro-peptide is essential for overexpression of soluble TGase in *E. coli *[9,11,17,18]. To investigate the effects of the secretory signal peptide and the covalently linked pro-peptide on expression of *S. hygroscopicus *TGase in *E. coli*, the pTG encoding TGase alone, the pSTG encoding TGase fused with the pelB signal peptide, and the pSPTG encoding pro-TGase fused with the pelB signal peptide were constructed and used for TGase expression (Figure 1A). As indicated by SDS-PAGE analysis, the TGase protein (38 kDa) was not expressed using the pTG (Figure 2A, lane 2, 4). However, the pSTG gave a high yield of the insoluble enzyme (Figure 2A, lane 1, 3). This result indicated that fusion of the signal peptide improved the expression of TGase. It is possible that the nascent peptide of TGase is sensitive to protease digestion in the cytoplasm [19]. The pro-TGase (45 kDa) was well expressed and secreted by *E. coli *harboring pSPTG (Figure 2A, lane 5-6). The secreted pro-TGase can be easily transformed into active TGase by dispase *in vitro *(data not shown), as described in our previous report [12]. These findings demonstrated that the covalently linked pro-peptide enhanced the solubility and secretion of the TGase in *E. coli*. However, reliance on protease-mediated activation *in vitro *would be unfavorable to the industrial application of the enzyme.

### Co-expression in the order of TGase and pro-peptide

Yurimoto et al. [16] have showed that active TGase could be produced directly by co-expression with its pro-peptide in methylotrophic yeast, suggesting that the pro-peptide mediated the correct folding of TGase in an inter-molecular manner. In order to examine the possibility of producing active TGase in *E. coli*, co-expression vectors were constructed on the rationale that the TGase pro-peptide and TGase should be driven by a single regulated promoter. A *lac *operator binding site was placed downstream of the T7 promoter with a ribosome-binding site preceding each gene. As illustrated in Figure 1B, the pro-peptide fused with or without the pelB signal peptide was cloned into the pTG in downstream of the TGase to generate pTG-PRO or pTG-SPRO. In addition, the pSTG-PRO and pSTG-SPRO were obtained by modifying pSTG in the same way (Figure 1B). Transcription and translation are coupled with translation beginning during mRNA synthesis in prokaryotes, and thus the order of transcription determines that of translation [20]. Four co-expression vectors described here permitted that the expression of TGase was prior to that of pro-peptide in *E. coli*.

As shown in Figure 2B (lanes 1, 2) and Figure 2C (lanes 1, 2), the TGase protein was still undetectable in both soluble and insoluble fractions of *E. coli *cells expressing pTG-PRO or pTG-SPRO. The TGase fused with the pelB signal peptide remained insoluble in *E. coli *cells expressing pSTG-PRO or pSTG-SPRO (Figure 2C, lanes 3-4). These results suggested that the expression of TGase was not improved by co-expression with pro-peptide in the order of the TGase and pro-peptide, regardless of the presence or absence of the secretory signal peptide. It has been reported that the order of expression may play a role in reconstitution of a protein complex in *E. coli *at high efficiency [2]. It is likely that altering the expression order of the TGase and pro-peptide had an effect on their interaction and subsequent benefit to the correct folding of the TGase in *E. coli*.

### Co-expression in the order of pro-peptide and TGase

In order to alter the expression order, we constructed four vectors (pPRO-TG, pPRO-STG, pSPRO-TG, and pSPRO-STG) in which the co-expression was initiated in the order of pro-peptide and TGase (Figure 1B). Similarly, the effect of the secretory signal peptide on TGase expression was taken into consideration. In pPRO-TG, the pro-peptide and TGase were co-expressed in the absence of the pelB signal peptide. In contrast, both pro-peptide and TGase were fused with the signal peptide in pSPRO-STG. The pelB signal peptide was merely fused to the TGase in pPRO-STG whereas the pro-peptide was fused with the signal peptide in pSPRO-TG. In line with the co-expression using the pTG-PRO and pTG-SPRO, the TGase was also not expressed by *E. coli *cells harboring pPRO-TG or pSPRO-TG (Figure 2BC, lanes 5-6). As yet unknown reason, the TGase fused with the pelB signal peptide was not produced by *E. coli *carrying pPRO-STG (Figure 2BC, lane 7). However, surprisingly soluble TGase was expressed by *E. coli *cells carrying pSPRO-STG (Figure 2B, lane 8) and distinct TGase activity (0.13 U/ml/OD_600_) was detected in the cell lysates of this strain (Figure 2D). Fusion of pelB signal peptide and prior expression of pro-peptide may help the proper folding of the TGase in *E. coli*. Protein aggregation usually follows a nucleation-polymerization mechanism, in which the buildup of the initial self-assembled nuclei constitutes the rate-limiting step of the reaction [21]. Therefore, the TGase is probably prone to nucleation in *E. coli*, and the early-expressed pro-peptide exclusively inhibits the nucleation of the nascent TGase peptide.

### Simultaneous co-expression of pro-peptide and TGase

To confirm the importance of the prior expression of pro-peptide, an additional T7 promoter was added in front of TGase gene in the pSPRO-STG to construct the pSPROT7STG which will permit simultaneous expression of pro-peptide and TGase in *E. coli*. As indicated by SDS-PAGE analysis, the addition of T7 promoter only induced insoluble TGase (Figure 2C, lane 9) than improving the yield of soluble TGase (Figure 2B, lane 9). The simultaneous expression of pro-peptide and TGase may have disrupted their required expression order, resulting in the incorrect folding.

### Translocation of the recombinant pro-TGase and TGase in *E. coli*

Amino acid sequencing of the recombinant TGase (MDAADE) revealed that the recombinant TGase produced by pSPRO-STG and native TGase had an identical N-terminus (from the second amino acid), suggesting that the fused signal peptide was correctly cleaved from the recombinant TGase. As the processing of the secretory signal peptide occurs at the periplasmic side of *E. coli *inner membrane [22], the recombinant TGase may have been transported into the periplasm after the co-expression. However, unlike the expression of pro-TGase fused with the pelB signal peptide (Figure 2A, lane 5), fusion of the signal peptide at N-terminal of the TGase failed to mediate its secretion by *E. coli *cells harboring pSPRO-STG (data not shown). Since non-specific periplasmic leakage is an important pathway for protein secretion in *E. coli *[23], the one most likely explanation for this discrepancy is related to the the cell permeability differences of the two expression systems. In our previous study, expression of TGase in active form has thicken the cell wall in *Lactococcus lactis *by crosslinking, and translocation of a short peptide into the cell was subsequently inhibited [24]. In this work, it was very difficult to extract the active TGase from the *E. coli *periplasm (data not shown) using a traditional method [11]. Active TGase produced by the co-expression may also resulted in thickening of the cell wall of *E. coli *that could impede the secretion of TGase. When the pro-TGase was expressed in *E. coli*, the TGase activity was completely inhibited by the covalently linked pro-peptide.

### Purification and characterization of the recombinant TGase

**Figure 3:**
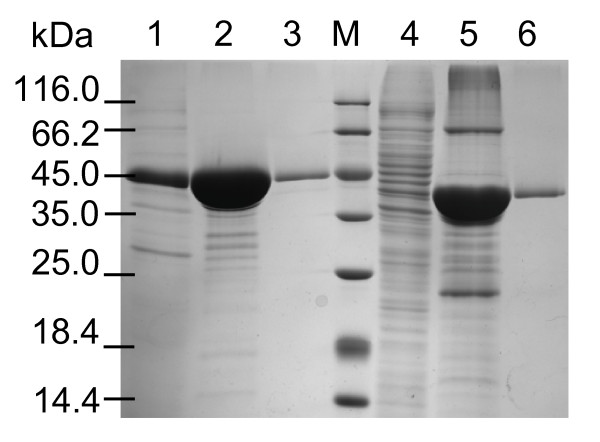
**SDS-PAGE analysis of purified recombinant pro-TGase and TGase**. 1: supernatants of *E. coli *cells harboring pSPTG; 2, 3: active fractions of nickel-affinity and the following gel-filtration chromatography of supernatants of *E. coli *cells harboring pSPTG, respectively; 4: intracellular soluble fraction of *E. coli *cells harboring pSPRO-STG; 5, 6: active fractions of nickel-affinity and the following gel-filtration chromatography of the intracellular soluble fraction of *E. coli *cells harboring pSPRO-STG, respectively.

After fusing with a His-tag at C-terminus, the pro-TGase and TGase expressed by *E. coli *cells harboring pSPTG and pSPRO-STG were purified to homogeneity using Ni^+ ^affinity chromatography and subsequent gel filtration (Figure 3). The purified pro-TGase was fully active after cleavage of pro-peptide *in vitro*. The native TGase purified from *S. hygroscopicus *was used to compare with the two recombinant TGases for the catalytic properties. As shown in Table 1, the optimal temperature and pH were identical for the three enzyme preparations (Table 1). In addition, the specific activity and kinetic parameter were similar for all the enzyme preparations tested (Table 1).

**Table 1:** Comparison of the catalytic properties of native and recombinant TGases.


**Resource**	**Optimal temperature** **(°C)**	**Optimal pH**	**Specific activity** **(U/mg)**	**K_m_** **(mmol/L)**

Native	40	6.0	18.2	54.7
^a^pSPTG	40	6.0	21.0	57.1
pSPRO-STG	40	6.0	22.0	56.6

^a ^Pro-TGase was transformed into active TGase through the addition of dispase.

### Insight into the folding pathway of TGase in *E. coli*

**Figure 4:**
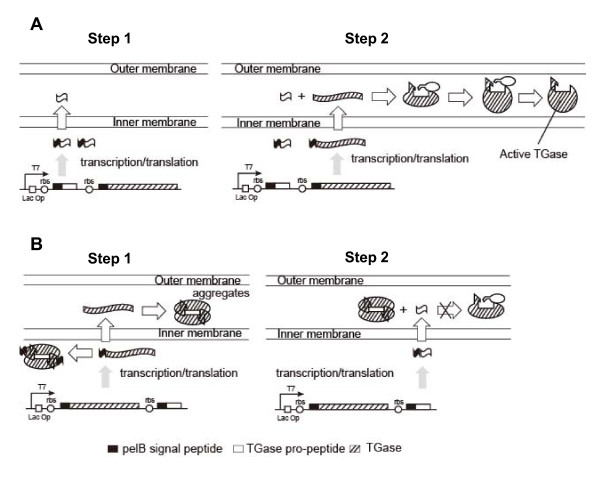
**A proposed mature pathway of TGase by co-expression in *E. coli***. (A) The co-expression in the order of TGase pro-peptide and TGase. (B) The co-expression in the order of TGase and its pro-peptide.

Based on above analysis, we propose a mature pathway of *Streptomyces *TGase assisted by the pro-peptide in *E. coli*. The pro-peptide and TGase were sequentially expressed under the same T7 promoter and exported into the periplasm by the pelB signal peptide (Figure 4A, step1). In the periplasm, the pro-peptide assisted the proper folding of the later-exported TGase which gained catalytic activity after releasing from the pro-peptide (Figure 4A, step2). When expressed and exported in prior to the pro-peptide, the TGase folded incorrectly resulting in aggregation in the cytoplasm or periplasm (Figure 4B).

## Conclusions

In conclusion, we have described a novel co-expression strategy for producing active TGase in *E. coli*. The recombinant *E. coli *yielded up to 0.13 U/ml/OD_600 _of the recombinant TGase that was comparable to the level of *E. coli *co-expressing pro-TGase with protease [14]. As the activating protease was not over-expressed, the co-expression strategy described here is well suited for the TGase production or as a molecular modification platform of the enzyme. In addition, the co-expression strategy based on optimizing the order of gene expression could be useful in the expression of other functional proteins that are synthesized as a precursor [25].

## Methods

### Construction of plasmids for TGase expression

*S. hygroscopicus *WSH03-13 that secretes TGase was isolated in a previous study [26]. *S. hygroscopicus *genomic DNA was isolated as described previously [27]. The primers for amplification of TGase-related gene were designed according to the *S. hygroscopicus *TGase gene sequence (GenBank ID: HM231108), which was previously published by our group [12]. The pro-TGase gene was cloned into the *Nco*I-*Xho*I site of pET-22b(+) (Novogen, Ontario, Canada) to yield pSPTG (Figure 1A).

The gene of TGase pro-peptide was first cloned into the *Nde*I-*Bam*HI and *Nco*I-*Bam*HI sites of pET-22b(+) to generate pPRO and pSPRO, respectively. The DNA sequences encoding the pro-peptide including the bacterial ribosome-binding site was amplified from the pPRO by PCR. The DNA sequences encoding the pro-peptide, pelB signal peptide gene, and ribosome-binding site was obtained from pSPRO by the same procedure. The two PCR procedures were performed using the following primer pair: 5'-ATA TGA GGA TCC GGA TAA CAA TTC CCC TCT AGA AAT AA-3' (P11) and 5'-GAC GAT GAA TTC TTA GGG GGC CCG GAA GAG-3' (P12).

The TGase gene was first cloned into the *Nde*I-*Bam*HI site and *Nco*I-*Bam*HI site of pET-22b(+) to generate pTG and pSTG, respectively (Figure 1A). The DNA sequences composed of TGase gene and bacterial ribosome-binding site were amplified from pPTG by PCR. The DNA sequences containing TGase gene, pelB signal peptide gene, and bacterial ribosome-binding site were amplified from pSTG by PCR. Both PCR procedures used the following primer pair: 5'-ATA TGA GGA TCC GGA TAA CAA TTC CCC TCT AGA AAT AA -3' (P21) and 5'-GAC GAT GAA TTC CGA CCA GCC CTG CTT CAC-3' (P22). The DNA sequence encoding TGase, pelB signal peptide, the ribosome-binding site, and T7 promoter was also amplified from pSTG using the primer 5'-TAA GAT CTC GAT CCC GCG A-3' (P31) and P22.

The resulting PCR products were cloned into the *Bam*HI-*Eco*RI site of pPRO, pSPRO, pTG, or pSTG to create the following co-expression plasmids: pTG-PRO, pTG-SPRO, pSTG-PRO, pSTG-SPRO, pPRO-TG, pPRO-STG, pSPRO-TG, pSPRO-STG, and pSPROT7STG (Figure 1B).

### Expression of pro-TGase and TGase in *E. coli*

The pro-TGase and TGase were expressed in *E. coli *BL21(DE3) (Novogen, Ontario, Canada). After the seed culture was grown in Luria-Bertani medium containing carbenicillin (50 μg/ml) at 37°C for 12 h, it was similarly grown in Terrific Broth medium containing carbenicillin (50 μg/ml) until the optical density at 600 nm reached 1.5. Isopropyl-β-D-thiogalactopyranoside was added to a final concentration of 0.4 mmol/L. After incubation for another 30 h at 20°C, the culture supernatant and cells were separated by centrifugation (10, 000 g, 5 min).

*E. coli *cells (5/OD_600_) harvested were sonicated in 500 μl of Tris-HCl buffer (pH 8) and then centrifuged (10, 000 g, 5 min). The supernatant was used for the analysis of the intracellular soluble fraction. The cell debris, corresponding to the intracellular insoluble fraction, was re-suspended in 500 μl of Tris-HCl buffer containing 1% SDS.

### Protein analysis

The purification of TGase from *S. hygroscopicus *culture was performed as previously described [28]. The purification of the recombinant enzyme (pro-TGase and TGase) was conducted using nickel affinity chromatography (HisTrap™ FF crude, GE Healthcare) and gel filtration (Superdex™75 10/300 GL, GE Healthcare). Dispase-mediated activation of pro-TGase was performed as previously described [13]. Protein content and SDS-PAGE analysis were conducted as previously described [28]. TGase activity was determined using N-CBZ-Gln-Gly at pH 6.0 and 37°C [28]. Amino acid sequencing of TGase N-terminal region was performed by Shanghai Gene Core Biotechnologies Co., Ltd.

## Competing interests

The authors declare that they have no competing interests.

## Authors' contributions

SL and DZ carried out the molecular genetic studies, and drafted the manuscript. MW, WC, and KC participated in the design of the study. GD, JC, and ZZ conceived of the study, and participated in its design and coordination and helped to draft the manuscript. All authors read and approved the final manuscript.
